# SARS-CoV-2 epidemiology, antibody dynamics, and neutralisation capacity in Irish healthcare workers in the era of booster COVID-19 vaccinations

**DOI:** 10.3389/fmed.2023.1078022

**Published:** 2023-01-26

**Authors:** Jonathan McGrath, Claire Kenny, Charlotte Salgaard Nielsen, Lisa Domegan, Cathal Walsh, Peadar Rooney, Shane Walsh, Niall Conlon, Gareth Brady, Aya Ibrahim, Jean Dunne, William McCormack, Niamh Corcoran, Niamh Allen, Catherine Fleming, Colm Bergin

**Affiliations:** ^1^Department of Genitourinary Medicine and Infectious Diseases (GUIDe), St. James’s Hospital, Dublin, Ireland; ^2^Department of Infectious Diseases, University Hospital Galway, Galway, Ireland; ^3^European Centre for Disease Prevention and Control (ECDC) Fellowship Programme, Field Epidemiology Path (EPIET), Solna, Sweden; ^4^Health Protection Surveillance Centre (HPSC), Dublin, Ireland; ^5^Department of Immunology, St. James’s Hospital, Dublin, Ireland; ^6^Department of Clinical Medicine, Trinity College, Dublin, Ireland; ^7^Trinity College, Trinity Health Kidney Centre, Trinity Translational Medicine Institute, St. James’s Hospital, Dublin, Ireland; ^8^Department of Clinical Medicine, Trinity Translational Medicine Institute, School of Medicine, Trinity College Dublin, Dublin, Ireland

**Keywords:** SARS-CoV-2, healthcare workers (HCW), antibody, neutralisation, variant, seroprevalence, vaccination, booster

## Abstract

**Background:**

The PRECISE Study, a multi-phase cross-sectional seroprevalence study of anti-SARS-CoV-2 antibodies in Irish healthcare workers (HCW) investigated: (1) risk factors for SARS-CoV-2 seropositivity, (2) the durability of antibody responses in a highly vaccinated HCW cohort, and (3) the neutralisation capacity of detected antibodies, prior to booster COVID-19 vaccination.

**Materials and methods:**

Serology samples were collected across two hospital sites in November 2021 and analysed using the Roche Elecsys Anti-SARS-CoV-2/Elecsys-S Anti-SARS-CoV-2 assays to detect anti-nucleocapsid (N) and anti-spike (S) antibodies respectively. Paired serology results from prior study phases were used to analyse changes in individual HCW serostatus over time. Risk-factors for SARS-CoV-2 infection were assessed for demographic and work-related factors. Antibody neutralisation capacity was assessed in a subset of samples *via* an *in vitro* ACE2 binding enzyme-linked immunosorbent assay.

**Results:**

2,344 HCW samples were analysed. Median age was 43 years (IQR 33–50) with 80.5% (*n* = 1,886) female participants. Irish (78.9%, *n* = 1,850) and Asian (12.3%, *n* = 288) were the most commonly reported ethnicities. Nursing/midwifery (39.3%, *n* = 922) was the most common job role. 97.7% of participants were fully vaccinated, with Pfizer (81.1%, *n* = 1,902) and AstraZeneca (16.1%, *n* = 377) the most common vaccines received. Seroprevalence for anti-SARS-CoV-2 antibodies indicating prior infection was 23.4%, of these 33.6% represented previously undiagnosed infections. All vaccinated participants demonstrated positive anti-S antibodies and in those with paired serology, no individual demonstrated loss of previously positive anti-S status below assay threshold for positivity. Interval loss of anti-N antibody positivity was demonstrated in 8.8% of previously positive participants with paired results. Risk factors for SARS-CoV-2 seropositivity suggestive of previous infection included age 18–29 years (aRR 1.50, 95% CI 1.19–1.90, *p* < 0.001), India as country of birth (aRR 1.35, 95% CI 1.01–1.73, *p* = 0.036), lower education level (aRR 1.35, 95% CI 1.11–1.66, *p* = 0.004) and HCA job role (aRR 2.12, 95% CI 1.51–2.95, *p* < 0.001). Antibody neutralisation varied significantly by anti-SARS-CoV-2 antibody status, with highest levels noted in those anti-N positive, in particular those with vaccination plus previous SARS-CoV-2 infection.

**Conclusion:**

All vaccinated HCWs maintained anti-S positivity prior to COVID-19 booster vaccination, however anti-N positivity was more dynamic over time. Antibody neutralisation capacity was highest in participants with COVID-19 vaccination plus prior SARS-CoV-2 infection.

## Introduction

Healthcare workers (HCW) are at increased risk of Severe Acute Respiratory Syndrome Coronavirus-2 (SARS-CoV-2) infection in comparison to the general community (1–3). Several factors have been identified that increase the risk of infection in HCW, including healthcare assistant (HCA) job role, nursing (4, 5), ethnicity (6–10), regular exposure to known patients with COVID-19 (3, 4), lack of access to PPE (1, 3, 4) and engagement in aerosol producing procedures (11). Conversely, intensive care unit (ICU) roles have been associated with lower likelihood of infection (6).

Infection with SARS-CoV-2 results in the development of both anti-nucleocapsid (N) and anti-spike (S) antibodies (12), while COVID-19 vaccines result in the production of anti-S antibodies alone (13). The SARS-CoV-2 spike (S) protein mediates viral entry into the host cell *via* the host ACE2 receptor and is a critical target for neutralising antibodies (NAb). NAb play a key role in primary prevention of infection and viral clearance (14). Crucial targets within the S-protein include the receptor binding domain (RBD) and N-terminal domain (NTD) on the S1 subunit (14). Accurate determination of virus neutralisation is challenging owing to the requirement for biosafety level 3 (BSL3) facilities utilising live SARS-CoV-2 viral models and as a result, surrogate lentiviral vectors pseudotyped with the SARS-CoV-2 spike protein are often adopted (14).

The Irish COVID-19 vaccination programme commenced on December 29th 2020 (15), with the additional/booster dose programme commencing in September 2021 (16). Robust antibody response and clinical protection has been demonstrated for the currently available COVID-19 vaccines following primary vaccination schedules with vaccine efficacies reported to range from 66 to 95% one to two months following completion of dosing (17–20). Statistical modelling based on vaccine efficacy trial data has suggested that vaccine-derived NAb levels are predictive of protection against both symptomatic and severe infection with SARS-CoV-2 (21).

As the pandemic has progressed, concerns have been raised regarding durability of antibody responses and duration of vaccine protection. As a result, booster dosing programmes have been implemented in many countries. Real-world vaccine effectiveness (VE) studies have demonstrated robust protection with BNT162b2 (Pfizer) vaccination 2–6 months following the second dose but have found evidence of considerable waning of immunity after 6 months, with VE declining to between 22 and 69% (22). Both total anti-SARS-CoV-2 antibodies (23) and vaccine-derived NAb titres (21) have been demonstrated to reduce over time but the reported magnitude and consequence of this reduction varies. A clear correlation between specific antibody titres and long-term VE remains unclear.

Five variants of concern (VOC) have been identified in Ireland up to date–B.1.1.7 (Alpha), B.1.351 (Beta), P.1 (Gamma), B.1.617.2 (Delta), and B.1.1.529 (Omicron) (24). Infection with VOC can significantly impact VE, with protection against symptomatic infection with the Omicron variant following primary mRNA vaccination schedules as low as 10% being demonstrated (25), although protection significantly increases following booster dosing (25, 26).

The Prevalence of Antibodies to SARS-CoV-2 in Irish healthcare workers (PRECISE) Study is a multi-site cross-sectional seroprevalence study of anti-SARS-CoV-2 antibodies in Irish HCW. Previous phases of the study were undertaken in October 2020 (*n* = 5,788) prior to COVID-19 vaccine availability (10) and April 2021 (*n* = 5,085) following COVID-19 vaccination rollout (27). The PRECISE study enables investigation of changing HCW epidemiological risks for SARS-CoV-2 infection and antibody dynamics over time. The current study was undertaken immediately prior to HCW COVID-19 3rd dose booster vaccinations and aims to assess: (1) risk factors for SARS-CoV-2 seropositivity and risk changes over time, (2) the durability of antibody responses in a highly vaccinated HCW cohort, and (3) the neutralisation capacity of detected antibodies prior to booster vaccination.

## Materials and methods

### Study design and participant recruitment

This is a cross-sectional seroprevalence study of anti-SARS-CoV-2 antibodies in Irish HCW undertaken in two hospital sites from 10th to 23rd November 2021. All HCWs who participated in the previous PRECISE study phase in April 2021 were invited to participate. Enrolment in this group was offered regardless of vaccination history or intent to receive further COVID-19 vaccine doses. Participants were recruited *via* internal hospital communications, emails and text messages.

Written consent was obtained and participants completed a written questionnaire detailing demographic, work-related and COVID-19 infection/vaccination details. Participants were then invited to undergo phlebotomy providing serology samples for anti-SARS-CoV-2 antibodies prior to receipt of their booster vaccination dose, if they were opting to receive one. Study numbers were assigned to each serology sample and processed pseudonymously. Antibody serology results with interpretive information were texted to participants on an opt-out basis.

### Study sites

Hospital site 1 is a tertiary referral centre and the largest university teaching hospital in Ireland, located in Dublin city with approximately 4,700 staff. Hospital site 2 is a tertiary referral centre in Galway city, with approximately 4,400 staff and serves as the main referral centre for Galway County.

### Laboratory analysis

All serology samples were analysed using the Roche Elecsys Anti-SARS-CoV-2 and Elecsys-S Anti-SARS-CoV-2 assays to detect total (IgG, A, M) anti-nucleocapsid (N) and anti-spike (S) antibodies respectively. Manufacturer’s reference ranges were used following an in-house verification process (28).

In participants with no history of vaccination, a positive anti-S and/or anti-N antibody result was considered evidence of previous SARS-CoV-2 infection. In participants with a history of vaccination, anti-S positivity alone was considered indicative of vaccination and anti-N positivity considered evidence of previous SARS-CoV-2 infection. Paired serology results from the April 2021 study phase were used to analyse changes in individual HCW serostatus over time.

The presence of neutralising antibodies capable of blocking interaction between spike protein-RBD and ACE2 was investigated in a subset of samples representative of the larger cohort, *via* an *in vitro* ACE2 binding enzyme-linked immunosorbent assay (ELISA). Methods of sample selection are detailed in Supplementary material A, section ii). This surrogate neutralisation assay demonstrates the extent to which biotinylated ACE2 interaction with spike-RBD is inhibited by antibodies in participant sera and has demonstrated close correlation with spike-based pseudo-virus infection assays for the assessment of ACE2-RBD binding neutralisation. Full details of this assay including comparator results to other assays are published elsewhere (29), see also Supplementary material A for further details.

### Statistical analysis

Frequencies and percentages were calculated for participant characteristics for the total study population and by hospital site. Comparisons between hospital sites were made using the Chi-square test. The prevalence of SARS-CoV-2 seropositivity was calculated by participant characteristics for the total study population and by hospital. Univariable and multivariable log-binomial regression provided crude and adjusted relative risks (RR) respectively, with their 95% confidence intervals (CI) in order to assess the association between participant characteristics and SARS-CoV-2 seropositivity. Following univariable analysis, variables with a *p*-value < 0.05 were considered in the multivariable analysis. Following a sensitivity analysis demonstrating consistent estimates when analysing only records with complete variables, multivariable analysis was carried out on 2,144 complete cases, with a total of 199 cases omitted due to missing values. Stepwise forward selection of variables was followed and variable addition assessed by analysis of variance (ANOVA) to determine improvement of model fit.

Comparison of median antibody titres between study phases was undertaken using the Mann-Whitney *U* test for variables with two levels and the Kruskal-Wallis test with Dunn’s multiple correction test for multi-level variables. Participant characteristics in the ACE2-RBD binding sub-group were compared to the larger cohort *via* Chi Square test. The Mann-Whitney *U* test and Kruskal-Wallis test with Dunn’s multiple test correction was used to compare the ACE2-RBD neutralisation observed by participant characteristics. All analyses were carried out using R [version 4.1.3, R Core Team, 2021 (30)] and GraphPad Prism (Version 9.4.1).

### Ethical approval

Ethical approval for this study was granted by the National Research Ethics Committee (NREC), application number 20-NREC-COV-101-AMEND-2.

## Results

### Study participation

A total of 2,415 HCWs were recruited and provided serology samples across the two study sites. Seventy-one participants were excluded due to having already received a booster vaccination at time of sampling, resulting in a population of 2,344 HCW included in the final analysis. By hospital site, 1,778 (75.9%) HCWs participated in Hospital site 1 and 566 (24.1%) in Hospital site 2, with 94% of participants having also taken part in the April 2021 PRECISE study phase.

### Demographics

Participant characteristics are detailed in Table 1a. Age, sex, and level of education were similar between hospital sites. Female participants accounted for 80.5% (*n* = 1,886) and median age was 43 years (IQR 33–50). Irish (Caucasian) ethnicity (78.9%, *n* = 1,850) was most commonly reported followed by Asian ethnicity (12.3%, *n* = 288) and Other White background (7.1%, *n* = 167). African or Other Black ethnicity was reported in 1.3% (*n* = 31). Ethnicity and country of birth differed between hospital sites. Nursing was the most common job role reported (39.3%, *n* = 922), followed by Allied Health (22.1%, *n* = 517) and administration roles (15.4%, *n* = 362), with some differences in job role representation between sites noted (Table 1a).

**TABLE 1a:** Participant characteristics by hospital and total study population, PRECISE 4, Ireland, November 2021.

Participant characteristics		Hospital site 1		Hospital site 2		*P*-value*	Total	
		(*N* = 1,778)		(*N* = 566)			(*N* = 2,344)	
		*n*	%	*n*	%		*n*	%
Age (year) Age groups (years)	Mean (SD)	41.8 (11.2)		42.2 (10.3)		-	41.9 (11.0)	
	Median (IQR)	42 (32–50)		43 (34–50)			43 (33–50)	
	18–29	311	17.5	74	13.1	**0.03**	385	16.4
	30–39	433	24.4	152	26.9		585	25.0
	40–49	533	30.0	192	33.9		725	30.9
	Over 50	501	28.2	148	26.1		649	27.7
Sex	Female	1,414	79.5	472	83.4	0.050	1,886	80.5
	Male	364	20.5	94	16.6		458	19.5
Ethnicity	Irish (white)	1,374	77.3	476	84.1	**<0.001**	1,850	78.9
	Any other white background	116	6.5	51	9.0		167	7.1
	Asian background	263	14.8	25	4.4		288	12.3
	African and other black background	23	1.3	8	1.4		31	1.3
	Unknown	2	0.1	6	1.1		8	0.3
Country of birth	Ireland	1,297	73.0	439	77.5	**<0.001**	1,736	74.0
	Philippines	127	7.1	4	0.7		131	5.6
	India	120	6.7	9	1.6		129	5.5
	United Kingdom	71	4.0	36	6.4		107	4.6
	Other	163	9.2	78	13.8		241	10.3
Education	Primary	3	0.2	0	0.0	0.299	3	0.1
	Secondary	192	10.8	53	9.4		245	10.5
	Third level	973	54.7	341	60.2		1,314	56.1
	Post-graduate	471	26.5	146	25.8		617	26.3
	Missing	139	7.8	26	4.6		165	7.0
Role	Administration	260	14.6	102	18.0	**0.019**	362	15.4
	Medical/Dental	154	8.6	44	7.8		198	8.5
	Nursing/Midwifery	709	39.9	213	37.6		922	39.3
	Allied health	396	22.3	121	21.4		517	22.1
	General support	110	6.2	25	4.4		135	5.8
	Healthcare assistant	79	4.4	25	4.4		104	4.4
	Other	42	2.4	27	4.8		69	2.9
	Missing	28	1.6	9	1.6		37	1.6

Values are *n* and % unless otherwise indicated. IQR, interquartile range; SD, standard deviation.

*Calculated using the chi-square test.

Bold values indicate significant *P*-values.

### COVID-19 and vaccination status

COVID-19 and vaccine-related participant characteristics are presented in Table 1b. For the total study cohort, 20.3% (*n* = 475) reported previous SARS-CoV-2 infection *via* self-reported positive SARS-CoV-2 PCR. Of these, 81.7% (*n* = 388) occurred prior to receipt of any COVID-19 vaccine dose (natural infection), 3.6% (*n* = 17) representing breakthrough infection (BI) following a single vaccine dose and 14.7% (*n* = 70) representing breakthrough infection post completion of primary vaccination schedules. The median time between completion of primary vaccination schedules and infection was 202 days (range 5–287 days).

**TABLE 1b:** COVID-19 related characteristics by hospital and total study population, PRECISE 4, Ireland, November 2021.

Participant characteristics		Hospital site 1		Hospital site 2		*P*-value*	Total	
		(*N* = 1,778)		(*N* = 566)			(*N* = 2,344)	
		*n*	%	*n*	%		*n*	%
Previous positive COVID-19 PCR test	Yes	353	19.9	122	21.6	0.414	475	20.3
	No	1,425	80.1	444	78.4		1,869	79.7
Seropositive^†^	Yes (seropositive)	429	24.1	119	21.0	0.266	548	23.4
	No (seronegative)	1,348	75.8	447	79.0		1,795	76.6
	Unknown	1	0.1	0	0.0		1	0.0
Undiagnosed infections^‡^	Yes (undiagnosed)	163	9.1	21	3.7	**<0.001**	184	7.8
	No (diagnosed)	1,614	90.8	545	96.3		2,159	92.1
	Unknown	1	0.1	0	0.0		1	0.1
Vaccination status	Vaccinated (any dose)	1,759	98.9	554	97.9	0.106	2,313	98.7
	Unvaccinated	18	1.0	12	2.1		30	1.2
	Unknown	1	0.1	0	0.0		1	0.1
Vaccine brand	Pfizer	1,421	79.9	481	85.0	**<0.001**	1,902	81.1
	AstraZeneca	322	18.1	55	9.7		377	16.1
	Other	15	0.8	17	3.0		32	1.4
	None (unvaccinated)	18	1.0	12	2.1		30	1.3
	Unknown	2	0.1	1	0.2		3	0.1
Vaccine type	mRNA (Pfizer, Moderna)	1,434	80.7	495	87.5	**<0.001**	1,929	82.3
	Viral vector (AstraZeneca, Janssen)	323	18.2	57	10.1		380	16.2
	Heterologous doses^¶^	1	0.1	1	0.2		2	0.1
	None (unvaccinated)	18	1.0	12	2.1		30	1.3
	Unknown	2	0.1	1	0.2		3	0.1

COVID-19, coronavirus disease-19; PCR, polymerase chain reaction.

*Calculated using the chi-square test.

^†^A participant was classified as seropositive if (1) unvaccinated and anti-spike (S) antibody plus anti-nucleocapsid (N) antibody positive or anti-S antibody positive alone or anti-N antibody positive alone, or (2) vaccinated and anti-S antibody plus anti-N antibody positive.

^‡^Undiagnosed infections were defined as seropositive with no self-reported previous laboratory-confirmed (by PCR) COVID-19 infection.

^¶^Heterologous doses refer to participants who received different COVID-19 vaccine brands/types for the first and second dose. Bold values indicate significant *P*-values.

High vaccination rates within the cohort were reported with 98.7% (*n* = 2,313) having received at least one vaccine dose (of whom, 97.7% had completed a primary vaccination schedule), 1.2% (*n* = 30) being unvaccinated and 0.1% (*n* = 1) with unknown vaccination status. Pfizer was the most common vaccine received (81.1%, *n* = 1,902) followed by AstraZeneca (16.1%, *n* = 377), with 1.4% (*n* = 32) receiving other vaccine brands. Where dates of vaccination were known (*n* = 1,839) the median time from completion of primary vaccination schedules to serology sampling was 298 days (range 39–312, IQR 281–300).

### SARS-CoV-2 seroprevalence

SARS-CoV-2 seroprevalence for the total study cohort and by hospital site is detailed in Table 2. Seroprevalence of SARS-CoV-2 antibodies for the total study cohort in November 2021 was 23.4% (*n* = 548) [24.1% Hospital site 1, 21.0% Hospital site 2, (Supplementary Tables 4, 5, Supplementary material B)]. Of these participants, 33.6% did not report a prior positive SARS-CoV-2 PCR, representing undiagnosed infections, accounting for 7.8% of the total study population. The highest crude SARS-CoV-2 seroprevalence was noted in the following groups: age group 18–29 years (30.6%, 95% CI: 26.2–35.4), African or other Black background ethnicity (32.3%, 95% CI: 18.1–50.6), India as country of birth (32.6%, 95% CI: 25.0–41.1) and primary level education (66.7%, 95% CI: 9.5–97.4). By job role, HCAs had the highest seroprevalence in both hospital sites (42.3%, 95% CI: 33.2–52.0), however prevalence by job role varied between sites (Table 2).

**TABLE 2:** Prevalence of SARS-CoV-2 seropositivity by participant characteristics, both hospitals, PRECISE 4, Ireland, November 2021 (*N* = 2,343)*.

Participant characteristics		Total	SARS-CoV-2 seropositivity		
		*N*	*n*	% (95% CI)	*P*-value^†^
Overall		2,343	548	23.4 (21.7–25.1)	–
Hospital	Hospital site 1	1,777^‡^	429	24.1 (22.2–26.2)	0.419
	Hospital site 2	566	119	21.0 (17.9–24.6)	
Age groups (years)	18–29	385	118	30.6 (26.2–35.4)	**0.002**
	30–39	584	122	20.9 (17.8–24.4)	
	40–49	725	160	22.1 (19.2–25.2)	
	Over 50	649	148	22.8 (19.7–26.2)	
Sex	Female	1,885	436	23.1 (21.3–25.1)	0.590
	Male	458	112	24.5 (20.7–28.6)	
Ethnicity	Irish (white)	1,850	403	21.8 (20.0–23.7)	**0.012**
	Any other white background	167	50	29.9 (23.5–37.3)	
	African and other black background	31	10	32.3 (18.1–50.6)	
	Asian background	287	83	28.9 (24.0–34.4)	
	Unknown	8	2	25.0 (5.7–65.0)	
Country of birth	Ireland	1,736	379	21.8 (19.9–80.1)	**0.011**
	Philippines	131	39	29.8 (22.5–38.2)	
	India	129	42	32.6 (25.0–41.1)	
	United Kingdom	107	21	19.6 (13.1–28.3)	
	Poland	33	9	27.3 (14.7–45.0)	
	USA	14	2	14.3 (3.4–44.1)	
	Other	193	56	29.0 (23.0–35.8)	
Education	Primary	3	2	66.7 (9.5–97.4)	**<0.001**
	Secondary	245	78	31.8 (26.3–37.9)	
	Third level	1,314	320	24.4 (22.1–26.8)	
	Post-graduate	617	104	16.9 (14.1–20.0)	
	Missing	164	44	26.8 (20.6–34.1)	
Role	Administration	362	70	19.3 (15.6–23.7)	**<0.001**
	Medical/Dental	198	38	19.2 (14.3–25.3)	
	Nursing/Midwifery	922	246	26.7 (23.9–29.6)	
	Allied health	517	89	17.2 (14.2–20.7)	
	General support	134	36	26.9 (20.0–35.0)	
	Healthcare assistant	104	44	42.3 (33.2–52.0)	
	Other	69	17	24.6 (15.8–36.2)	
	Missing	37	8	21.6 (11.1–37.9)	

CI, confidence interval; NA, not applicable; SARS-CoV-2, severe acute respiratory syndrome coronavirus 2.

*A participant was classified as seropositive if (1) unvaccinated and anti-spike (S) antibody plus anti-nucleocapsid (N) antibody positive or anti-S antibody positive alone or anti-N antibody positive alone, or (2) vaccinated and anti-S antibody plus anti-N antibody positive.

^†^Calculated using the chi-squared test.

^‡^Seropositivity unknown for one subject.

Bold values indicate significant *P*-values.

### Factors associated with seropositivity

The results of the univariable and multivariable analysis are presented in Table 3. Following multivariable analysis for the total study population, the adjusted RR (aRR) for seropositivity was significant for the following participant characteristics: age 18–19 years (aRR 1.50, 95% CI: 1.19–1.90, *p* < 0.001), India as country of birth (aRR 1.35, 95% CI: 1.01–1.73, *p* = 0.036), education level–primary/secondary/third (post-graduate education as referent) (aRR 1.35, 95% CI: 1.11–1.66, *p* = 0.004) and HCA job role (aRR 2.12, 95% CI: 1.51–2.95, *p* < 0.001). Analysis by hospital site demonstrated some variability in relation to job role, with medical/dental staff (aRR 2.80, 95% CI: 1.30–6.39, *p* = 0.01), nursing or midwifery (aRR 2.47, 95% CI: 1.35–5.19, *p* = 0.007), and general support staff (aRR 2.94, 95% CI: 1.09–7.33, *p* = 0.021) associated with seropositivity in Hospital site 2, but not in Hospital site 1 (Supplementary Tables 6, 7, Supplementary material B).

**TABLE 3:** Association between risk factors and SARS-CoV-2 seropositivity, PRECISE 4, Ireland, November 2021 (*N* = 2,343).

Participant characteristics		*n*	Unadjusted relative risk (95% CI)	*P*-value	Adjusted relative risk (95% CI) (complete case analysis, *N* = 2,144)	*P*-value
Hospital	Hospital site 2	566	Ref.			
	Hospital site 1	1,777*	1.15 (0.96–1.38)	0.130		
Age groups (years)	18–29	385	1.34 (1.09–1.65)	**<0.001**	1.52 (1.21–1.90)	**<0.001**
	30–39	584	0.92 (0.74–1.12)	0.420	1.01 (0.81–1.27)	0.906
	40–49	725	0.97 (0.79–1.18)	0.740	1.05 (0.85–1.29)	0.679
	Over 50	649	Ref.		Ref.	
Sex	Female	1,885	Ref.			
	Male	458	1.06 (0.88–1.26)	0.550		
Ethnicity^‡^	Irish (white)	1,850	Ref.			
	Any other white background	167	1.37 (1.06–1.74)	**0.010**		
	Asian background	287	1.37 (1.06–1.74)	**0.010**		
	African and other black background	31	1.48 (0.81–2.30)	0.140		
	Unknown	8	1.15 (0.21–2.73)	0.820		
Country of birth	Ireland	1,736	Ref.		Ref.	
	Philippines	131	1.36 (1.01–1.77)	**0.030**	1.17 (0.85–1.57)	0.307
	India	129	1.49 (1.12–1.91)	**<0.001**	1.35 (1.01–1.73)	**0.035**
	United Kingdom	107	0.90 (0.58–1.29)	0.600	0.93 (0.59–1.35)	0.725
	Other	240	1.28 (1.01–1.58)	**0.030**	1.12 (0.87–1.42)	0.348
Education (*N* = 2,179)	Primary/Secondary/Third level	1,562	1.52 (1.26–1.86)	**<0.001**	1.35 (1.11–1.66)	**0.004**
	Post-graduate	617	Ref.		Ref.	
Role (*N* = 2,306)	Administration	362	Ref.		Ref.	
	Medical/Dental	198	0.99 (0.69–1.40)	0.970	0.96 (0.66–1.39)	0.350
	Nursing/Midwifery	922	1.38 (1.10–1.76)	**0.010**	1.29 (0.99–1.69)	0.059
	Allied health	517	0.89 (0.67–1.18)	0.420	0.87 (0.64–1.18)	0.350
	General support	134	1.39 (0.97–1.95)	0.070	1.28 (0.84–1.89)	0.224
	Healthcare assistant	104	2.19 (1.60–2.96)	**<0.001**	2.12 (1.51–2.95)	**<0.001**
	Other	69	1.27 (0.77–1.96)	0.310	1.21 (0.70–1.92)	0.457
Vaccination status (any dose)^‡^	Vaccinated (any dose)	2,313	Ref.			
	Unvaccinated	30	2.17 (1.41–2.95)	**<0.001**		
Vaccine brand (*N* = 2,341)^‡^	Pfizer	1,902	0.79 (0.66–0.95)	**0.010**		
	AstraZeneca	377	Ref.			
	Other	32	1.93 (1.27–2.67)	**<0.001**		
	None (unvaccinated)	30	1.81 (1.15–2.57)	**<0.001**		
Vaccine type (*N* = 2,341)^‡^	mRNA (Pfizer, Moderna)	1,929	0.78 (0.66–0.95)	**0.010**		
	Viral vector (AstraZeneca, Janssen)	380	Ref.			
	Heterologous doses^†^	2	^†^			
	None (unvaccinated)	30	1.78 (1.13–2.51)	**<0.001**		

CI, confidence interval; SARS-CoV-2, severe acute respiratory syndrome coronavirus 2.

*Seropositivity unknown for one subject.

^†^Excluded from analysis due to small number in category.

^‡^Excluded from analysis due to small numbers in some categories and resulting multicollinearity in multivariable analysis. Bold values indicate significant P-values.

### Antibody durability and serostatus dynamics over time

All vaccinated participants demonstrated positive anti-S antibodies. No individual with paired sera (*n* = 2,208) demonstrated loss of anti-S antibody positivity below the assay threshold for positivity between April and November 2021. In the paired sera cohort, 1,880 (80.2%) participants had anti-S antibody titre levels > 250 U/mL (the upper limit of quantification for the commercial assay) in November 2021 compared with 1,829 (82.8%) in April 2021.

**FIGURE 1:**
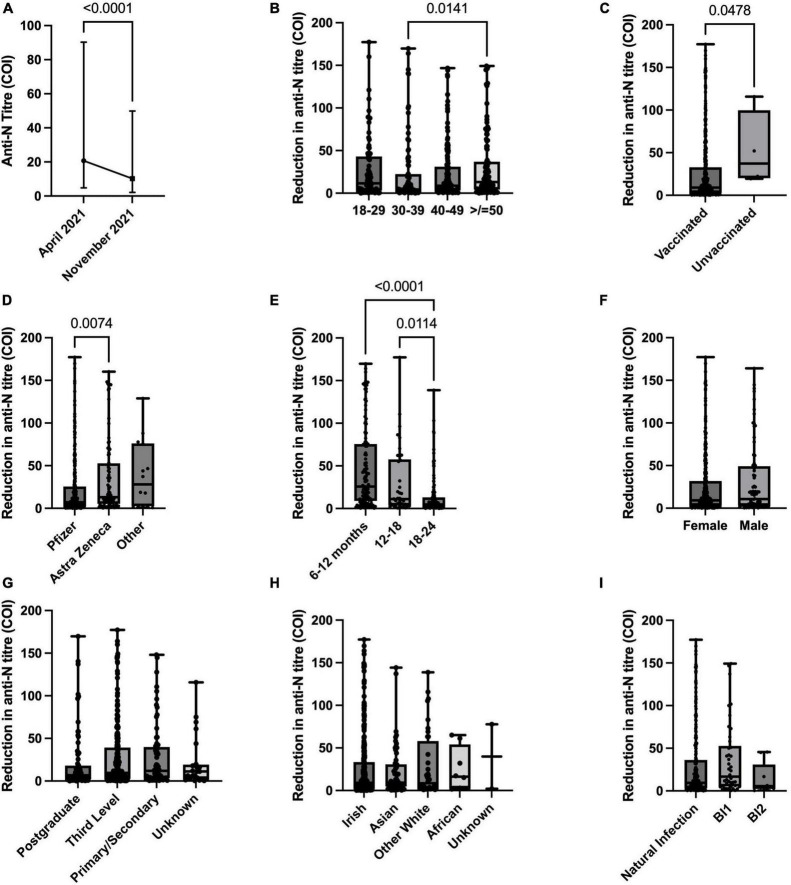
Anti-nucleocapsid (anti-N) titre dynamics over time between April 2021 and November 2021. **(A)** Anti-N titre changes for paired sera cohort with positive anti-N in April 2021. B-I: Reduction in anti-N titres by **(B)** age group, **(C)** vaccination status, **(D)** COVID-19 vaccine received, **(E)** time from reported positive SARS-CoV-2 positive PCR to study serology sampling, **(F)** sex, **(G)** education level, **(H)** ethnicity, **(I)** infection status–natural (pre-vaccine), breakthrough following one vaccine dose (BI1), breakthrough following second vaccine dose (BI2).

Anti-N antibodies were more dynamic over time with 8.8% (*n* = 40) of previously anti-N positive individuals (*n* = 457) losing positive status between April and November 2021. There was a significant decrease in anti-N titres for the cohort as a whole between the two study phases with median values of 23.4 COI (cut-off index) (range 1–262, IQR 6.8–70.9) in April 2021 decreasing to median 10.9 COI (range 0.271–330, IQR 2.79–34.3, *p* < 0.0001) in the intervening 7-months period (November 2021) (Figure 1). By age group, those aged 50 years or older (*n* = 107) had a significantly greater reduction in titres in comparison to those aged 30–39 years (*n* = 92) (reduction of median 13.15 vs. 4.365 COI, *p* = 0.014), with no other significant differences demonstrated amongst other age groups (Figure 1). Unvaccinated HCWs (*n* = 4) demonstrated greater reductions in titres in comparison to those vaccinated (*n* = 407) (median decrease 37.2 vs. 9.13 COI, *p* = 0.047) and HCWs vaccinated with the AstraZeneca vaccine (*n* = 88) demonstrated greater titre reductions in comparison to those in receipt of the Pfizer vaccine (*n* = 304) (median decrease 12.97 vs. 7.13 COI, *p* = 0.007). Amongst participants anti-N positive in April 2021, dates for positive PCR results were known for 62.6% (*n* = 286). Of these, time since infection impacted reduction in anti-N titres, with those infected 18–24 months prior to the current study demonstrating significantly smaller reductions in titres than those infected more recent to the study period, at 12–18 months or 6–12 months prior to study sampling (median reduction 4.8 vs. 11.1 (*p* = 0.01) and 25.7 (*p* < 0.001) COI respectively). No significant differences in titre reductions were noted in relation to sex, ethnicity, education level or infection status (natural versus breakthrough infection). Anti-N positivity was demonstrated in 70.6% (*n* = 12) of individuals with BI between dose 1 and 2 and in 72.9% (*n* = 51) of those with BI post second dose. Forty-five (9.8%) individuals increased their anti-N titres. Of these, where positive PCR dates were known (*n* = 29), SARS-CoV-2 infections occurred median 412 days (range 271–619, IQR 323–590) prior to the current study period.

### ACE2-RBD binding assay cohort–Antibody neutralisation

#### Demographics and vaccination status

Demographic, COVID-19 and vaccine related characteristics for the ACE2-RBD binding assay sub-cohort are listed in Supplementary material A. Ninety samples were tested using the *in vitro* ACE2-RBD binding ELISA. Age, sex, ethnicity, country of birth, education, and vaccine type received did not vary significantly from the total cohort, although differences in job role and vaccination status proportions were noted.

#### ACE2-RBD binding inhibition

**FIGURE 2:**
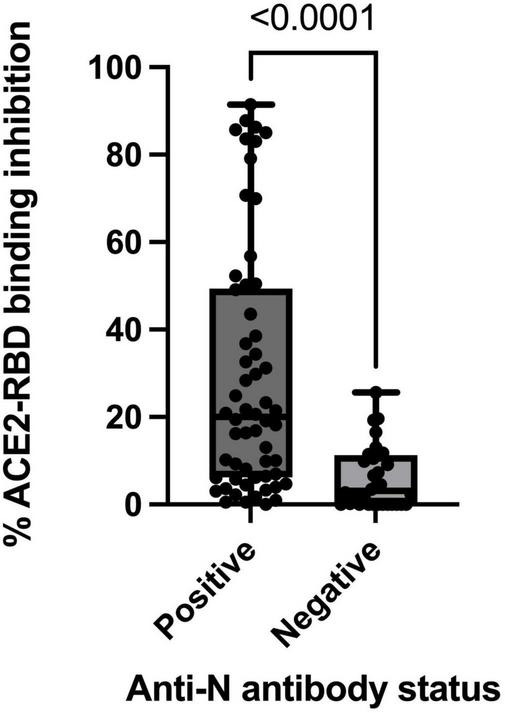
ACE2-RBD protein-protein binding inhibition stratified by participant anti-nucleocapsid antibody status for total sub-cohort (*n* = 90).

The extent of ACE2-RBD binding inhibition varied significantly by SARS-CoV-2 antibody status (Figure 2). The median ACE2-RBD inhibition demonstrated in anti-N positive individuals was 19.4% (mean, 28.9%, range 1–91%, *n* = 57) compared to 3.1% (mean 6%, range 0–25%, *n* = 32) in those anti-N negative (*p* < 0.0001) (Figure 2). Within the anti-N positive group, vaccinated participants demonstrated the highest levels of ACE2-RBD binding inhibition with median 20.9% (mean 30.5%, range 0–91, *n* = 53) compared to those unvaccinated with median 5.3% (mean 7.3%, range 0.5–18.2, *n* = 4). Within the anti-N positive group, where a positive PCR was identified, the median time from PCR to study sampling was 379.5 days (IQR 307–590, *n* = 34). When assessed according to anti-N antibody status, (anti-N positive or anti-N negative), ACE2-RBD protein-protein inhibition did not vary significantly by age, sex or type of vaccine received (Pfizer or Astra Zeneca).

The median ACE2-RBD binding inhibition was 13% in natural infection (mean 24.3%, range 0–87.8, *n* = 30), a median 415 days from infection to sampling (range 22–623 days, IQR 309.25–591.5) and was higher in those with vaccination plus natural infection (median 16.2%, mean 25.8%, range 0–87.8%, *n* = 27) compared to those with natural infection alone (7.0, 11.5, and 13% respectively, *n* = 3). ACE2-RBD inhibition was median 21.7% in those with breakthrough infection (*n* = 3, mean 16.1%) following the first vaccine dose (median 309 days from infection to sampling) and median 46.8% in those with infection following the second vaccine dose (*n* = 4) (median 85 days from infection to sampling).

Participants in receipt of the Pfizer vaccine, without evidence of prior SARS-CoV-2 infection, demonstrated a median ACE2-RBD inhibition of 1.7% (*n* = 21, mean 4.5%, range 0–19.6%), after a median 298 days from second dose (range 267–307 days, IQR 295–301). Participants in receipt of the AstraZeneca vaccine, without evidence of prior SARS-CoV-2 infection, demonstrated ACE2-RBD inhibition of median 3.5% (*n* = 5, mean 9.7%, range 0–25.6%) after a median 196 days from second dose (range 179–202 days, IQR 190–196). ACE2-RBD inhibition in vaccine recipients with anti-N positivity was median 21.6% for Pfizer vaccine recipients (*n* = 46, mean 32.6%) and median 12.8% for AstraZeneca vaccine recipients (*n* = 6, mean 16.5%).

## Discussion

We present the findings of a multi-site, multi-phase epidemiological and seroprevalence cross-sectional study of SARS-CoV-2 infection in a defined at-risk cohort. Longitudinal analysis of a HCW population allows the assessment of risk dynamics over time in addition to identifying changes in serostatus within subgroups that may inform decisions regarding local infection prevention and control (IPC) measures and regarding future vaccination strategies. Multi-phase seroprevalence studies reflect cumulative risk for infection over time and thus incident infections within subgroups are additive to the previously noted prevalence, however significant interval increases within subgroups were noted. Waning of immunity and diminishing protection from primary COVID-19 vaccination schedules has been a growing concern during the pandemic, particularly with the emergence of VOC. Serological sampling in this study occurred immediately prior to HCW booster dosing and therefore provides an accurate assessment of the serostatus of a large cohort prior to the influence of additional booster doses.

The SARS-CoV-2 antibody seroprevalence in the total PRECISE cohort was 23.4% in November 2021, increased from 18% in April 2021, and 10% in October 2020. This reflects the magnitude of the fourth pandemic wave of COVID-19 in Ireland (31). Hospital site 1 had a higher seroprevalence at 24.1% in comparison to Hospital site 2 at 21%, likely impacted by the varying COVID-19 incidence rates in the more densely populated Dublin county in comparison to Galway county (759.8 vs. 553 per 100,000 population for Week 47, 2021) (31).

The October 2020 phase of the PRECISE study identified SARS-CoV-2 seroprevalence rates in HCWs six times higher than that of the general community (10). The magnitude of this differential was less pronounced in the current study, with community SARS-CoV-2 seroprevalence (assessed *via* anti-N positivity) at the time of study sampling estimated at 12.1% (32), just under half of that seen in the PRECISE HCW study. This finding demonstrates the increasing importance of community SARS-CoV-2 transmission as the pandemic has progressed, yet also demonstrates the persistent increased risks experienced by HCW for infection.

Of the SARS-CoV-2 infections identified serologically, 33.6% were previously undiagnosed. Reported rates of undiagnosed infections with SARS-CoV-2 vary, with estimates between 26 and 40% reported (10, 27, 33). The findings here demonstrate that despite evolving testing protocols throughout the pandemic, a significant proportion of infections go unidentified, possibly through lack of access to testing and/or pauci-/asymptomatic presentations. Institutional IPC measures should reflect this prevalence and ensure robust testing procedures are in place, especially during COVID-19 outbreaks, acknowledging that current testing based on development of symptoms or arising through COVID-contact identification may fail to identify a significant proportion of cases. The emergence of the Omicron variant soon after study sampling further emphasises this issue, as asymptomatic infection rates of 32.4% have been reported with this VOC (34).

Individuals within the age category 18–29 years were identified as higher risk for seropositivity, consistent with prior PRECISE phases and other studies (4, 27). Individuals with primary level of education were noted to have the highest crude SARS-CoV-2 seroprevalence within the cohort at 67%, an increase of 34.7% from April 2021. This increase was demonstrated in Hospital site 1, as no HCW in Hospital site 2 reported primary level of education alone. Lower level of education was also identified as a factor associated with seropositivity on multivariable analysis, with the combined variable of primary/secondary/tertiary educated HCWs having higher risk for seropositivity than post-graduate educated participants. The underlying cause of this may be a combination of social and work-related factors. Lower levels of education are often associated with lower socio-economic status which has been noted to increase both risk of and poorer outcomes with SARS-CoV-2 infection (35). Local IPC policies must acknowledge this persisting at-risk group and ensure that messaging is appropriate for a wide audience and accessible *via* various means/media to ensure that modifiable work-related risks for transmission are addressed where possible.

Our findings demonstrate that HCW reporting India as country of birth were at increased risk of SARS-CoV-2 seropositivity. Seropositivity rates within this group for the total cohort was determined at 33%, with a significant interval increase from April 2021 (24% to 56%) noted in Hospital site 2. Ethnicity has been identified as a risk factor for HCW SARS-CoV-2 infection with South Asian (9, 10), Black (6–8) and Hispanic (4) ethnicities frequently identified as being at increased risk, although the individual groups at highest risk throughout the pandemic has been dynamic (27). Due to issues relating to multi-collinearity with “country of birth,” “ethnicity” was not entered into the multivariable regression model. A signal is noted however on univariable analysis with Asian background and Any Other White Background identified as being associated with seropositivity in comparison to Irish, predominantly Caucasian, colleagues. This signal is consistent with the multivariable model finding given the high South Asian representation in participants from India. This finding may reflect a combination of factors including high representation in identified high-risk job roles (HCA/nursing, below) and additional non-work related factors including accommodation circumstances, access to healthcare, education, and biological factors within a given population (6).

Healthcare workers (HCW) job role is well-recognised as a factor associated with SARS-CoV-2 seropositivity, with HCA and nursing roles frequently identified as high risk (4, 5, 27). In our findings, HCAs demonstrated the highest crude seroprevalence rate (42%) amongst HCW job roles at both study sites, an increase of 10.3% from April ‘21. HCA job role was additionally associated with seropositivity following multivariable analysis. Risk for seropositivity amongst other HCW roles differed between the hospital sites, with medical/dental staff, nursing/midwifery and general support staff additionally demonstrating association *via* multivariable analysis in Hospital site 2 and not in Hospital site 1 (Supplementary material B). PPE use is a significant determinant of SARS-CoV-2 infection risk in HCW (36, 37) and notably PPE supplies were maintained in both sites throughout the pandemic without significant disruption. The variable risk demonstrated amongst job roles may reflect differing IPC/patient flow policies between the two institutions or other contributing factors not included in this study, such as shared accommodation and socialising among different HCW groups.

Significant vaccine uptake rates are demonstrated in our cohort with 98.7% of participants receiving at least one vaccine dose and 97.7% having completed a primary vaccination schedule. BNT162b2 (Pfizer, 81.1%) and ChAdOx1 (AstraZeneca, 16.1%) were the most common vaccines received, with primary vaccination schedules completed a median 298 days (IQR 281–300) prior to study sampling.

Reductions in anti-S-RBD IgG antibodies 6 months following Pfizer vaccination decreasing to 7% of their post-second-dose peak have been demonstrated, comparable to levels measured after the first vaccine dose or after SARS-CoV-2 natural infection (23). Levin et al. demonstrated significant waning of humoral responses, assessed *via* anti-S-RBD IgG titres, within 6 months after receipt of the second dose of the Pfizer vaccine in a large cohort of 4,868 HCWs (38). In our findings, 100% of vaccinated individuals demonstrated positive anti-S status. Additionally, no individual with paired sera demonstrated reduction of anti-S antibody titres (total IgG, A, M) below the assay threshold for positivity between April and November 2021. Mapping of individual anti-S titre variability was not possible due to the high proportion of participants in both the April 2021 (82.8%) and November 2021 (80.2%) study phases demonstrating anti-S antibody levels above the Roche Elecsys-S assay threshold for further quantification (> 250 U/mL). Qualitatively, persistence of anti-S positivity has been noted in convalescent sera prior to vaccine availability at varying time-points, with studies demonstrating anti-S IgG positivity of 90% at 8 months post symptom onset (39). Our findings demonstrate 100% anti-S positivity at more prolonged time-points, likely influenced by the high rates of vaccination in the HCW cohort.

Anti-N antibodies were more dynamic with 8.8% of individuals losing anti-N positivity over the intervening 7-month period. This reflects findings of other studies which have demonstrated greater reductions in anti-N titres over time in comparison to anti-S, with qualitative anti-N positivity of 76% noted 8 months post onset of disease (40). Lumley et al. reported that anti-N antibodies begin to decline within 1 month post positive PCR, with an estimated half-life of 85 days and reported that 50% of studied HCW became seronegative after 7 months (41), prior to the availability of vaccines; although reported anti-N half-lives vary amongst reports (42). In our findings, greater reductions in anti-N titres were noted with increasing age, in keeping with studies such as REACT2 (43), however some reports demonstrate higher peak anti-N titres and prolonged half-lives in older individuals, hypothesising cross-reactivity with other coronaviruses (41). The current study HCW cohort is of working age (median 43 years) and therefore a broader study demographic is likely required to further clarify this association. Time-since-infection impacts anti-N titres with an initial post-infection peak followed by decline, before low level stabilisation. Specific intervals in reported patterns vary, with one study citing anti-N titre rise until day 40 (D40) post infection, followed by decline until D120 and subsequently stable levels from D120 to D240 (40). A similar effect is reflected in our results with individuals infected closer to the study sampling period demonstrating greater interval decreases in titres compared to HCW with more distant infections, reflecting a stabilisation in titres over longer periods of time.

Unvaccinated individuals demonstrated greater decreases in anti-N titres than those vaccinated. The increased titres seen with vaccination, combined with the effect of time on titre reductions may have influenced titre variations demonstrated between Pfizer and AstraZeneca vaccines in our findings, as the latter vaccine was rolled out at a later date as part of the Irish vaccination programme. It has been noted that not all SARS-CoV-2 infections lead to development of an anti-N antibody response (40) and indeed only 70.6%/72.9% of individuals with BI between dose 1 and 2/post-2nd dose respectively mounted an anti-N response in our findings. Previous PRECISE study phases have also noted that in individuals with prior positive SARS-CoV-2 PCR results, only 82% demonstrated seropositivity (27). Loss of anti-N positivity or lack of seroconversion in a given population has epidemiological consequences as it may lead to the underestimation of infection exposure within a given individual or population over time, impacting interpretation of personal infection history or population-wide SARS-CoV-2 prevalence.

Given the key role Spike-RBD-ACE2 binding plays in viral entry into cells, evaluating the capacity of participant anti-S antibodies to block this protein-protein interaction *via* the *in vitro* ACE2-binding assay may provide a more effective indication of humoral protection than routine serology alone. Vaccine-derived NAb play an important protective role following vaccination, with neutralising antibody titres strongly correlated with protection from symptomatic infection (44), however this protection decreases with time (21). The RBD-ACE2 binding inhibition ELISA utilised in our study acts as a surrogate neutralisation marker, assessing this key protein-protein interaction.

Higher levels of RBD-ACE2 binding inhibition (median 20.9%) were demonstrated in participants with both vaccination and previous SARS-CoV-2 infection (evidenced by anti-N positivity) than those with either vaccination or infection alone, suggesting a greater degree of humoral immune protection in this subgroup. RBD-ACE2 binding inhibition of mean 4.5 and 9.7% was demonstrated for participants in receipt of Pfizer and AstraZeneca vaccination alone, a median 298 days and 196 days post second dose respectively. This is comparable to the inhibition seen in unvaccinated individuals with natural infection alone, determined *via* anti-N positivity, at 7.3% (*n* = 4). While inhibition was increased in the Astra Zeneca group compared to the Pfizer group, this is influenced by time interval from primary vaccination schedule completion to serology sampling (as above) and did not show statistically significant differences.

These observations are supported by findings in VE studies, with Hall et al. demonstrating VE consistently higher than 90% in those with infection plus vaccination up to 18 months following infection, while infection-acquired immunity waned in individuals with natural infection alone after 1 year (22). The degree of RBD-ACE2 binding inhibition required to mediate this increased protection in vaccinated/previously-infected individuals is unclear and is likely one factor of many in the functional immune protection conferred by vaccination. Additionally, it is noted that modelling data suggests that the neutralisation level required for protection from severe infection is sixfold lower than the level required to protect from any symptomatic infection (21) and thus the lower RBD-ACE2 inhibition levels demonstrated in the vaccination-alone/infection-alone subgroups may still provide a degree of protection over time.

The reported magnitude of NAb titre reductions over time varies, with some studies demonstrating a median decrease of 34.8% 3 months post vaccination (45). Our findings support a reduction in NAbs over time, with ACE2-RBD inhibition of median 46.8% demonstrated in participants with infection following the second vaccine dose a median 85 days prior to sampling, in contrast to 16.2% in individuals with natural infection and vaccination a median 415 days prior to sampling. Given the small numbers involved in these sub-groups in the current study, it is difficult to determine statistical significance from this finding but it should be considered a signal warranting further investigation.

The emergence of variants of concern (VOC) has resulted in reductions in vaccine effectiveness (25) and vaccine-derived NAbs (44). Reports have demonstrated significant NAb titre reductions against the Omicron variant in individuals in receipt of two doses of mRNA vaccine or with a history of natural infection alone without vaccination (46). Booster vaccine dosing with mRNA vaccines mitigates this antibody reduction with significant increases in titres demonstrated in both previously infected individuals and those in receipt of vaccination alone (44, 46). The variations in participant antibody neutralisation capacity demonstrated in our findings, assessed by infection/vaccination status, support findings identifying at-risk groups for VOC infection and add to the evidence and rationale for additional vaccine doses. As population-wide vaccine programmes become increasingly challenging logistically, a more targeted approach may be preferable with higher risk groups as identified above, being prioritised for further vaccinations.

### Limitations

The authors acknowledge a number of limitations with the current study. Due to issues related to multicollinearity, HCW “ethnicity” and “country of birth” could not both be included in the multivariable regression model and as such, aRR could not be determined for both variables. Similarly, due to the robust COVID-19 vaccine uptake amongst staff, reliable statistical conclusions could not be drawn regarding risk for seropositivity by vaccination status due to the small numbers of HCWs who remained unvaccinated. While HCWs from the “HCA,” “African/Other Black ethnicity,” and “India as country of birth” demonstrated high seroprevalence and/or significant risk for SARS-CoV-2 seropositivity as outlined above, a relatively small number of HCWs from each of these subgroups were sampled, representing 4.4, 1.3, and 5.5% of the total study cohort respectively. It is noted however that despite this under-representation, these subgroups have been consistently identified as high risk in a number of studies as outlined above. The upper limit of quantification for the Roche Elecsys-S antibody assay of 250 IU/mL made further quantification and tracking of anti-S titres above this level not logistically possible. A significant proportion of participants in both the current study phase and the April study phase demonstrated titres exceeding this limit and thus statistical titre comparisons were not possible. This limitation has been noted in other studies using commercial assays (41). Data in relation to participant co-morbidities or symptoms with prior infection was not available and thus antibody dynamics stratified by co-morbidity/symptomatology was not determined. The ACE2-RBD binding inhibition ELISA utilises a SARS-CoV-2 Spike protein-RBD based on the wild-type Wuhan Hu-1 strain and therefore the authors cannot comment specifically on the neutralisation capacity of measured antibodies against other VOC. Additionally, it is noted that the ELISA used will not identify NAbs that target sites other than RBD, for example those targeted toward the NTD.

## Conclusion

Healthcare workers (HCW) remain a high-risk population for SARS-CoV-2 infection with seroprevalence rates double that of the community rate at time of sampling. HCW age, job role, education level, and country of birth were determined to be factors associated with SARS-CoV-2 seropositivity. Local IPC measures should reflect the persistent increases in seroprevalence in these high-risk groups, acknowledging that despite approximately 2 years of communication regarding IPC practices and PPE protocols, certain job roles are persistently at increased risk. Increased efforts should be made using targeted messaging within these groups to mitigate modifiable risks within a clinical setting where possible.

The anti-N antibody dynamics demonstrated may impact future sero-epidemiological assessment of SARS-CoV-2 infection on both an individual and population-based level. Loss of anti-N positivity influencing assessment of prior exposure should be taken into consideration in future seroprevalence investigations. While all vaccinated individuals demonstrated persistence of anti-S positivity, the neutralisation capacity of these antibodies was influenced by vaccination and SARS-CoV-2 infection history. With the emergence of VOC and increasing logistical issues with population-wide vaccination, our findings support a more targeted approach toward future vaccine programmes with at-risk groups being prioritised for further dosing i.e., those with a history of vaccination or infection alone.

The impact that variable immune responses have on breakthrough SARS-CoV-2 infections, particularly in light of VOC and booster vaccine dosing continues to be elucidated. The PRECISE study group has undertaken an independent breakthrough infection study to investigate this variability.

## PRECISE Study Steering Group

Precise Study (Prevalence of COVID-19 in Irish Healthcare Workers) Steering Group Members and Affiliations: Dr. Lorraine Doherty, National Clinical Director for Health Protection, HSE-Health Protection Surveillance Centre (HPSC), Dublin, Ireland, and Chair of Steering Group, NA, Consultant Physician in Infectious Diseases, CB, Consultant Physician in Infectious Diseases and Site Lead for PRECISE study, St. James’s Hospital, Dublin, Ireland, NCon, Consultant Immunologist, St. James’s Hospital, Dublin, Ireland, LD, Surveillance Scientist, HSE-HPSC, Dublin, Ireland, CF, Consultant in Infectious Disease and Site Lead for PRECISE study, Galway University Hospital, Galway, Ireland, Dr. Margaret Fitzgerald, National Public Health Lead. National Social Inclusion Office, Dublin, Ireland, Dr. Cillian de Gascun, Director, UCD National Virus Reference Laboratory, University College Dublin, Dublin, Ireland, Joan Gallagher, Programme Manager, Office of the National Clinical Director for Health Protection, HSE-HPSC, Dublin, Ireland, Dr. Derval Igoe, Specialist in Public Health Medicine, HSE-HPSC, Dublin, Ireland, Prof. Mary Keogan, Consultant Immunologist Beaumont Hospital and Clinical Lead, National Clinical Programme for Pathology, HSE, Ireland, Dr. Noirin Noonan, Consultant in Occupation Medicine, St. James’s Hospital, Dublin, Ireland, Professor Cliona O’Farrelly, Chair in Comparative Immunology, Trinity College Dublin, Ireland, Dr. Una Ni Riain, Consultant Microbiologist, Galway University Hospital, Galway, Ireland, and Dr. Breda Smyth, Department of Public Health, HSE West, Ireland.

## Data availability statement

The raw data supporting the conclusions of this article will be made available by the authors, without undue reservation.

## Ethics statement

The studies involving human participants were reviewed and approved by National Research Ethics Committee (NREC). The patients/participants provided their written informed consent to participate in this study.

## Author contributions

JM and CK aided in study design, study execution, data analysis, and wrote the manuscript. CF and CB acted as site leads in each site and oversaw and contributed to the study design, execution, data analysis, and manuscript review. NA contributed to the study design and manuscript review. NCon, JD, GB, AI, and WM undertook laboratory analysis, contributed to the study design, and reviewed the manuscript. PR and SW contributed to the study design, study execution, data analysis, and reviewed the manuscript. CN, LD, and CW undertook data analysis and reviewed the manuscript. NCor aided in data collection, study execution, and reviewed the manuscript. The PRECISE Steering group aided in study design and study execution. All authors reviewed the final manuscript and approved the submitted version.
