# Supplementary material for: SARS-CoV-2 epidemiology, antibody dynamics, and neutralisation capacity in Irish healthcare workers in the era of booster COVID-19 vaccinations
Source: Front Med (Lausanne). 2023 Jan 26;10:1078022. doi: 10.3389/fmed.2023.1078022 (PMC9909389; doi:10.3389/fmed.2023.1078022)
Supplement: Supplementary file 1 [file Table_1.DOCX]

SARS-CoV-2 seroprevalence by hospital site

**Table 4** Prevalence of SARS-CoV-2 seropositivity by participant characteristics, Hospital Site 1, PRECISE 4, Ireland, November 2021*

| Participant characteristics | | Total | SARS-CoV-2 seropositivity | | |
| --- | --- | --- | --- | --- | --- |
|  |  | N | n | % (95% CI) | P-value^†^ |
| Overall |  | 1777 ^‡^ | 429 | 24.1 (22.2 - 26.2) | **-** |
| Age groups (years) | 18-29 | 311 | 100 | 32.2 (27.2 - 37.6) | **0.003** |
|  | 30-39 | 432 | 92 | 21.3 (17.7 - 25.4) |  |
|  | 40-49 | 533 | 122 | 22.9 (19.5 - 26.7) |  |
|  | Over 50 | 501 | 115 | 23.0 (19.5 -26.8) |  |
| Sex | Female | 1,413 | 339 | 24.0 (21.8 - 26.3) | 0.824 |
|  | Male | 364 | 90 | 24.7 (20.6 - 29.4) |  |
| Ethnicity | Irish (white) | 1,374 | 304 | 22.1 (20.0 - 24.4) | **0.003** |
|  | Any other white background | 116 | 36 | 31.0 (23.3 - 40.1) |  |
|  | Asian background | 262 | 78 | 29.8 (24.5 - 35.6) |  |
|  | African and other black background | 23 | 10 | 43.5 (24.9 - 64.1) |  |
|  | Unknown | 2 | 1 | 50.0 (1.9 - 98.1) |  |
| Country of birth | Ireland | 1,297 | 287 | 22.1 (19.9 - 24.5) | **0.03** |
|  | Philippines | 127 | 39 | 30.7 (23.3 - 39.3) |  |
|  | India | 120 | 37 | 30.8 (23.2 - 39.7) |  |
|  | United Kingdom | 71 | 16 | 22.5 (14.2 - 33.8) |  |
|  | Poland | 10 | 2 | 20.0 (4.6 - 56.2) |  |
|  | USA | 5 | 1 | 20.0 (2.1 - 74.4) |  |
|  | Other | 147 | 47 | 32.0 (24.9 - 40.0) |  |
|  | Delete row |  |  |  |  |
| Education | Primary | 3 | 2 | 66.7 (9.55 - 97.4) | **<0.001** |
|  | Secondary | 192 | 63 | 32.8 (26.5 - 39.8) |  |
|  | Third level | 973 | 252 | 25.9 (23.2 - 28.7) |  |
|  | Post-graduate | 471 | 76 | 16.1 (13.1 - 19.7) |  |
|  | Missing | 138 | 36 | 26.1 (19.4 - 34.1) |  |
| Role | Administration | 260 | 58 | 22.3 (17.6 - 27.8) | **<0.001** |
|  | Medical/dental | 154 | 25 | 16.2 (11.2 - 23.0) |  |
|  | Nursing/midwifery | 709 | 193 | 27.2 (24.1 - 30.6) |  |
|  | Allied health | 396 | 73 | 18.4 (14.9 - 22.6) |  |
|  | General support | 109 | 29 | 26.6 (19.1 - 35.7) |  |
|  | Healthcare assistant | 79 | 34 | 43.0 (32.5 - 54.2) |  |
|  | Other | 42 | 11 | 26.2 (15.0 - 41.6) |  |
|  | Missing | 28 | 6 | 21.4 (9.8 - 40.6) |  |

CI = confidence interval, NA = not applicable, SARS-CoV-2 = severe acute respiratory syndrome coronavirus 2

* A participant was classified as seropositive if (1) Unvaccinated **and** anti-spike (S) antibody plus anti-nucleocapsid (N) antibody positive **or** anti-S antibody positive alone **or** anti-N antibody positive alone, or (2) Vaccinated **and** anti-S antibody plus anti-N antibody positive.

^†^ Calculated using the chi-squared test. ^†^ Seropositivity unknown for one subject.

**Table 5** Prevalence of SARS-CoV-2 seropositivity by participant characteristics, Hospital Site 2, PRECISE 4, Ireland, November 2021*

| Participant characteristics | | Total | SARS-CoV-2 seropositivity | | |
| --- | --- | --- | --- | --- | --- |
|  |  | N | n | % (95% CI) | P-value^†^ |
| Overall |  | 566 | 119 | 21.0 (17.9 - 24.6) | **-** |
| Age groups (years) | 18-29 | 74 | 18 | 24.3 (15.8 - 35.5) | 0.812 |
|  | 30-39 | 152 | 30 | 19.7 (14.1 - 26.9) |  |
|  | 40-49 | 192 | 38 | 19.8 (14.7 - 26.1) |  |
|  | Over 50 | 148 | 33 | 22.3 (16.3 - 29.8) |  |
| Sex | Female | 472 | 97 | 20.6 (17.1 - 24.4) | 0.630 |
|  | Male | 94 | 22 | 23.4 (15.9 - 33.1) |  |
| Ethnicity | Irish (white) | 476 | 99 | 20.8 (17.4 - 24.7) | 0.478 |
|  | Any other white background | 51 | 14 | 27.5 (16.9 - 41.4) |  |
|  | African and other black background | 8 | 0 | NA |  |
|  | Asian background | 25 | 5 | 20.0 (8.4 - 40.5) |  |
|  | Unknown | 6 | 1 | 16.7 (1.8 - 67.9) |  |
| Country of birth | Ireland | 439 | 92 | 21.0 (17.4 - 25.0) | 0.106 |
|  | Philippines | 4 | 0 | NA |  |
|  | India | 9 | 5 | 55.6 (23.6 - 83.5) |  |
|  | United Kingdom | 36 | 5 | 13.9 (5.82 - 29.6) |  |
|  | Poland | 23 | 7 | 30.4 (15.0 - 52.1) |  |
|  | USA | 9 | 1 | 11.1 (1.3 -53.3) |  |
|  | Other | 46 | 9 | 19.6 (10.4 - 33.7) |  |
| Education | Primary | 0 | 0 | NA | 0.334 |
|  | Secondary | 53 | 15 | 28.3 (17.7 - 41.9) |  |
|  | Third level | 341 | 68 | 19.9 (16.0 - 24.5) |  |
|  | Post-graduate | 146 | 28 | 19.2 (13.6 - 26.4) |  |
|  | Missing | 26 | 8 | 30.8 (15.9 - 51.0) |  |
| Role | Administration | 102 | 12 | 11.8 (6.78 - 19.7) | **0.003** |
|  | Medical/dental | 44 | 13 | 29.5 (17.9 - 44.7) |  |
|  | Nursing/midwifery | 213 | 53 | 24.9 (19.5 - 31.2) |  |
|  | Allied health | 121 | 16 | 13.2 (8.24 - 20.6) |  |
|  | General support | 25 | 7 | 28.0 (13.7 - 48.7) |  |
|  | Healthcare assistant | 25 | 10 | 40.0 (22.7 - 60.2) |  |
|  | Other | 27 | 6 | 22.2 (10.2 - 41.9) |  |
|  | Missing | 9 | 2 | 22.2 (5.1 - 60.3) |  |

CI = confidence interval, NA = not applicable, SARS-CoV-2 = severe acute respiratory syndrome coronavirus 2

* A participant was classified as seropositive if (1) Unvaccinated **and** anti-spike (S) antibody plus anti-nucleocapsid (N) antibody positive **or** anti-S antibody positive alone **or** anti-N antibody positive alone, or (2) Vaccinated **and** anti-S antibody plus anti-N antibody positive.

^†^ Calculated using the chi-squared test.

Associations with SARS-CoV-2 seropositivity by study hospital site

**Table 6** Association between risk factors and SARS-CoV-2 seropositivity, Hospital site 1, PRECISE 4, Ireland, November 2021 (N=1,777*)

| Participant characteristics | | n | Unadjusted relative risk (95% CI) | P-value | Adjusted relative risk  (95% CI) (complete case analysis, N=1,614) | P-value |
| --- | --- | --- | --- | --- | --- | --- |
| Age groups (years) | 18-29 | 311 | 1.49 (1.11 - 1.76) | **<0.001** | 1.59 (1.24 - 2.04) | **<0.001** |
|  | 30-39 | 432 | 0.93 (0.73 - 1.18) | 0.540 | 1.05 (0.81 - 1.36) | 0.703 |
|  | 40-49 | 533 | 1.00 (0.80 - 1.25) | 0.980 | 1.10 (0.810 - 1.40) | 0.458 |
|  | Over 50 | 501 | Ref. |  | Ref. |  |
| Sex | Female | 1,413 | Ref. |  |  |  |
|  | Male | 364 | 1.03 (0.84 - 1.25) | 0.770 |  |  |
| Ethnicity ^‡^ | Irish (white) | 1,374 | Ref. |  |  |  |
|  | Any other white background | 116 | 1.25 (0.93 - 1.64) | 0.120 |  |  |
|  | Asian background | 262 | 1.37 (1.11 - 1.67) | **<0.001** |  |  |
|  | African and other black background | 23 | 1.53 (0.81 - 2.42) | 0.120 |  |  |
|  | Unknown | 2 | 1.31 (0.25 - 3.02) | 0.650 |  |  |
| Country of birth | Ireland | 1,297 | Ref. |  | Ref. |  |
|  | Philippines | 127 | 1.39 (1.03 - 1.81) | **0.020** | 1.20 (0.87 - 1.59) | 0.225 |
|  | India | 120 | 1.39 (1.02 - 1.82) | **0.020** | 1.27 (0.93 - 1.68) | 0.117 |
|  | United Kingdom | 71 | 1.02 (0.62 - 1.52) | 0.940 | 1.12 (0.67 - 1.68) | 0.614 |
|  | Other | 162 | 1.39 (1.07 - 1.77) | **0.010** | 1.16 (0.85 - 1.52) | 0.308 |
| Education (N=1,639) | Primary/secondary/third level | 1,168 | 1.68 (1.35 - 2.13) | **<0.001** | 1.48 (1.18 - 1.90) | **<0.001** |
|  | Post-graduate | 471 | Ref. |  | Ref. |  |
| Role (N=1,749) | Administration | 260 | Ref. |  | Ref. |  |
|  | Medical/dental | 154 | 0.73 (0.47 - 1.10) | 0.140 | 0.70 (0.44 - 1.08) | 0.115 |
|  | Nursing/midwifery | 709 | 1.22 (0.95 - 1.59) | 0.130 | 1.08 (0.82 - 1.45) | 0.601 |
|  | Allied health | 396 | 0.83 (0.61 - 1.13) | 0.220 | 0.78 (0.57 - 1.09) | 0.146 |
|  | General support | 109 | 1.19 (0.80 - 1.73) | 0.370 | 1.04 (0.64 - 1.59) | 0.868 |
|  | Healthcare assistant | 79 | 1.93 (1.36 - 2.69) | **<0.001** | 1.82 (1.24 - 2.60) | **0.001** |
|  | Other | 42 | 1.17 (0.63 - 1.95) | 0.570 | 1.24 (0.64 - 2.11) | 0.469 |
| Vaccination status (any dose) ^‡^ | Vaccinated (any dose) | 1,759 | Ref. |  |  |  |
|  | Unvaccinated | 18 | 3.05 (2.08 - 3.85) | **<0.001** |  |  |
| Vaccine brand (1,776) ^‡^ | Pfizer | 1,421 | 0.83 (0.69 - 1.03) | **<0.001** |  |  |
|  | AstraZeneca | 322 | Ref. |  |  |  |
|  | Other | 15 | 2.22 (1.27 - 3.23) | **<0.001** |  |  |
|  | None (unvaccinated) | 18 | 2.67 (1.77 - 3.59) | **<0.001** |  |  |
| Vaccine type (1,776) ^‡^ | mRNA (Pfizer, Moderna) | 1,434 | 0.84 (0.69 - 1.03) | 0.090 |  |  |
|  | Viral vector  (AstraZeneca, Janssen) | 323 | Ref. |  |  |  |
|  | Heterologous doses ^†^ | 1 | ^†^ |  |  |  |
|  | None (unvaccinated) | 18 | 2.65 (1.76 - 3.56) | **<0.001** |  |  |

CI = confidence interval, SARS-CoV-2 = severe acute respiratory syndrome coronavirus 2.
* Seropositivity unknown for one subject.
^†^ Excluded from analysis due to small number in category.
^‡^ Excluded from analysis due to small numbers in some categories and resulting multicollinearity in multivariable analysis.

**Table 7** Association between risk factors and SARS-CoV-2 seropositivity, Hospital Site 2, PRECISE 4, Ireland, November 2021 (N=566)

| Participant characteristics | | n | Unadjusted relative risk (95% CI) | P-value | Adjusted relative risk  (95% CI) (complete case analysis, N=530) | P-value |
| --- | --- | --- | --- | --- | --- | --- |
| Age groups (years) | 18-29 | 74 | 1.09 (0.65 - 1.78) | 0.730 | 1.23 (0.72 - 2.06) | 0.410 |
|  | 30-39 | 152 | 0.89 (0.57 - 1.38) | 0.590 | 0.93 (0.58 -1.48) | 0.765 |
|  | 40-49 | 192 | 0.89 (0.59 - 1.35) | 0.570 | 0.98 (0.64 - 1.52) | 0.945 |
|  | Over 50 | 148 | Ref. |  | Ref. |  |
| Sex | Female | 472 | Ref. |  |  |  |
|  | Male | 94 | 1.14 (0.74 - 1.67) | 0.530 |  |  |
| Ethnicity | Irish (white) | 476 | Ref. |  |  |  |
|  | Any other white background | 51 | 1.32 (0.77 - 2.04) | 0.260 |  |  |
|  | Asian background | 25 | 0.96 (0.36 - 1.89) | 0.920 |  |  |
|  | African and other black background | 8 | * |  |  |  |
|  | Unknown | 6 | * |  |  |  |
| Country of birth | Ireland | 439 | Ref. |  |  |  |
|  | Philippines | 4 | * |  |  |  |
|  | India | 9 | * |  |  |  |
|  | United Kingdom | 36 | 0.66 (0.25 - 1.35) | 0.330 |  |  |
|  | Other | 78 | 1.04 (0.63 - 1.59) | 0.870 |  |  |
| Education (N=540) | Primary/secondary/third level | 394 | 1.10 (0.76 -1.65) | 0.630 |  |  |
|  | Post-graduate | 146 | Ref. |  |  |  |
| Role (N=557) | Administration | 102 | Ref. |  | Ref. |  |
|  | Medical/dental | 44 | 2.51 (1.24 - 5.17) | **0.010** | 2.80 (1.29 - 6.39) | **0.010** |
|  | Nursing/midwifery | 213 | 2.12 (1.23 - 3.99) | **0.010** | 2.47 (1.35 - 5.19) | **0.007** |
|  | Allied health | 121 | 1.12 (0.56 - 2.32) | 0.740 | 1.26 (0.58 - 2.89) | 0.567 |
|  | General support | 25 | 2.38 (0.98 - 5.31) | **0.040** | 2.92 (1.08 - 7.28) | **0.022** |
|  | Healthcare assistant | 25 | 3.40 (1.62 - 7.03) | **<0.001** | 3.93 (1.73 - 9.04) | **<0.001** |
|  | Other | 27 | 1.89 (0.71 - 4.41) | 0.160 | 1.83 (0.60 - 4.87) | 0.242 |
| Vaccination status (any dose) ^†^ | Vaccinated (any dose) | 554 | Ref. |  |  |  |
|  | Unvaccinated | 12 | 0.79 (0.14 - 2.08) | 0.720 |  |  |
| Vaccine brand (N=565) ^†^ | Pfizer | 481 | 0.62 (0.41 - 1.00) | **0.030** |  |  |
|  | AstraZeneca | 55 | Ref. |  |  |  |
|  | Other | 17 | 1.52 (0.74 - 2.81) | 0.200 |  |  |
|  | None (unvaccinated) | 12 | 0.54 (0.09 - 1.56) | 0.360 |  |  |
| Vaccine type (N=565) ^†^ | mRNA (i.e. Pfizer, Moderna) | 495 | 0.59 (0.41 - 0.93) | **0.010** |  |  |
|  | Viral vector (i.e. AstraZeneca, Janssen) | 57 | Ref. |  |  |  |
|  | Heterologous doses * | 1 | * |  |  |  |
|  | None (unvaccinated) | 12 | 0.50 (0.09 - 1.43) | 0.300 |  |  |

CI = confidence interval, SARS-CoV-2 = severe acute respiratory syndrome coronavirus 2.
* Excluded from analysis due to small number in category.
^†^ Excluded from analysis due to small numbers in some categories and resulting multicollinearity in multivariable analysis.
