# Supplementary material for: SARS-CoV-2 epidemiology, antibody dynamics, and neutralisation capacity in Irish healthcare workers in the era of booster COVID-19 vaccinations
Source: Front Med (Lausanne). 2023 Jan 26;10:1078022. doi: 10.3389/fmed.2023.1078022 (PMC9909389; doi:10.3389/fmed.2023.1078022)
Supplement: Supplementary file 2 [file Table_2.DOCX]

*In vitro* ACE2-RBD Binding Inhibition ELISA

**i)**

Laboratory methodology for *in vitro* ACE2 Binding Assay

Methodology as published by Phelan et al. (1)

Ninety-six-well plates were coated with spike RBD and blocked. Serum samples were prepared in 1% milk PBST, serially diluted two-fold from a 1:50 dilution and left on the plates for 1 h at room temperature. A positive control was included in the form of a commercial IgG1 anti-spike antibody containing 2% normal human serum (Thermo Fisher Scientific, Waltham, MA, USA, MA5-35939). After washing three times with 0.1% PBST, 50 μL/well of 10 μg/mL biotinylated ACE2 was added to the plates and left for 1 h at room temperature. The ACE2 solution was removed and the plates washed three times. Streptavidin HRP was diluted 1:40 in PBS and 100 μL/well was added to the plate for 20 min. The streptavidin HRP was removed and the plates were washed three times. After washing, 100 μL SigmaFast OPD was added to each well for 10 min and the reaction was stopped using 3M hydrochloric acid. The optical density was measured at 492 nm using a Multiskan FC plate reader.

**ii)**

Sample selection for ACE2-RBD inhibition assay from larger sample set

The total Hospital site 1 serology samples were divided by anti-Spike antibody titre results into groups <50 IU/mL, >/=50 and </=250 IU/mL and >250 IU/mL as determined by the Roche Elecsys-S Anti-SARS-CoV-2 assay. Proportional numbers of samples were randomly selected from these subgroups to be analysed via the ACE2-RBD binding assay. While samples were not chosen based on demographic findings, the table in Section iii) below demonstrates that the resultant sub-cohort was closely representative of the larger Hospital site 1 cohort.

**iii)**

| **Participant characteristics in ACE2-RBD binding sub-cohort in comparison to total study population, PRECISE 4, Ireland, November 2021** | | | | | | |
| --- | --- | --- | --- | --- | --- | --- |
| Participant characteristics | | Total Study Cohort | | ACE2 Binding Subcohort | | P-value* |
|  |  | (N=2,344) | | (N=90) | |  |
|  |  | N | % | N | % |  |
| Age (years) | Mean (SD) | 41.9 (11.0) | | 44.3 (11.4) | |  |
|  | Median (IQR) | 43 (33 – 50) | | 45 (34 – 53) | |  |
| Age groups (years) | 18-29 | 385 | 16.4 | 13 | 14.4 | 0.15 |
|  | 30-39 | 585 | 25.0 | 19 | 21.1 |  |
|  | 40-49 | 725 | 30.9 | 22 | 24.4 |  |
|  | 50-59 | 523 | 22.3 | 29 | 32.2 |  |
|  | Over 60 | 126 | 5.4 | 7 | 7.8 |  |
| Sex | Female | 1,886 | 80.5 | 71 | 78.9 | 0.7 |
|  | Male | 458 | 19.5 | 19 | 21.1 |  |
| Ethnicity | Irish (white) | 1,850 | 78.9 | 69 | 76.7 | 0.9 |
|  | Any other white background | 167 | 7.1 | 6 | 6.7 |  |
|  | Asian background | 288 | 12.3 | 13 | 14.4 |  |
|  | African and other black background | 31 | 1.3 | 2 | 2.2 |  |
|  | Unknown | 8 | 0.3 | 0 | 0 |  |
| Country of birth | Ireland | 1,736 | 74.0 | 64 | 71.1 | 0.9 |
|  | Philippines | 131 | 5.6 | 6 | 6.7 |  |
|  | India | 129 | 5.5 | 7 | 7.8 |  |
|  | United Kingdom | 107 | 4.6 | 4 | 4.4 |  |
|  | Other | 241 | 10.3 | 9 | 10 |  |
| Education | Primary | 3 | 0.1 | 0 | 0 | 0.2 |
|  | Secondary | 245 | 10.5 | 16 | 17.8 |  |
|  | Third level | 1,314 | 56.1 | 50 | 55.6 |  |
|  | Post-graduate | 617 | 26.3 | 20 | 22.2 |  |
|  | Missing | 165 | 7.0 | 4 | 4.4 |  |
| Role | Administration | 362 | 15.4 | 14 | 15.6 | **<0.001** |
|  | Medical/dental | 198 | 8.5 | 5 | 5.6 |  |
|  | Nursing/midwifery | 922 | 39.3 | 36 | 40 |  |
|  | Allied health | 517 | 22.1 | 12 | 13.3 |  |
|  | General support | 135 | 5.8 | 14 | 15.6 |  |
|  | Healthcare assistant | 104 | 4.4 | 8 | 8.9 |  |
|  | Other | 69 | 2.9 | 0 | 0 |  |
|  | Missing | 37 | 1.6 | 1 | 1.1 |  |
| Vaccination status | Vaccinated (any dose) | 2,313 | 98.7 | 82 | 91.1 | **<0.001** |
|  | Unvaccinated | 30 | 1.2 | 8 | 8.9 |  |
|  | Unknown | 3 | 0.1 | 0 | 0 |  |
|  | mRNA  (Pfizer, Moderna) | 1,929 | 82.3 | 70 | 85.4 | 0.6 |
|  | Viral vector (AstraZeneca, Janssen) | 380 | 16.2 | 11 | 13.4 |  |
| * P-values determined via Chi-square test. | | | | | | |

1. Phelan T, Dunne J, Conlon N, Cheallaigh CN, Abbott WM, Faba-Rodriguez R, et al. Dynamic Assay for Profiling Anti-SARS-CoV-2 Antibodies and Their ACE2/Spike RBD Neutralization Capacity. Viruses. 2021;13(7).
